# Ivermectin-induced fixed drug eruption in an elderly Cameroonian: a case report

**DOI:** 10.1186/s13256-018-1801-1

**Published:** 2018-09-11

**Authors:** Calypse Asangbe Ngwasiri, Martin Hongieh Abanda, Leopold Ndemnge Aminde

**Affiliations:** 1 Bamendjou District Hospital, Bamendjou, West Region Cameroon; 2 Clinical Research Education, Networking & Consultancy (CRENC), Douala, Cameroon; 3 School of Public Health, Faculty of Medicine, The University of Queensland, Brisbane, Australia

**Keywords:** Fixed drug eruption, Ivermectin, Onchocerciasis

## Abstract

**Background:**

Cutaneous adverse reactions to medications are extremely common and display characteristic clinical morphology. A fixed drug eruption is a cutaneous adverse drug reaction due to type IV or delayed cell-mediated hypersensitivity. Ivermectin, a broad-spectrum anti-parasitic compound, has been an essential component of public health campaigns targeting the control of two devastating neglected tropical diseases: onchocerciasis (river blindness) and lymphatic filariasis.

**Case presentation:**

We report the case of a 75-year-old Cameroonian man of the Bamileke ancestry who developed multiple fixed drug eruptions a few hours following ivermectin intake that worsened with repeated drug consumption. Discontinuation of the drug, counselling, systemic steroids, and orally administered antihistamines were the treatment modalities employed. Marked regression of the lesions ensued with residual hyperpigmentation and dyschromia.

**Conclusion:**

Keen observation on the part of physicians is mandatory during the administration of ivermectin for quick recognition and prevention of this adverse drug reaction.

## Background

A fixed drug eruption (FDE) is a cutaneous adverse drug reaction (CADR) due to type IV or delayed cell-mediated hypersensitivity [1]. It can occur even when some drugs are used within normal doses and describes the development of one or more annular or oval erythematous patches as a result of systemic exposure to that drug [2]. These lesions typically resolve spontaneously after discontinuation of the causative agent but leave a residual hyperpigmentation at the reaction sites. Repeated exposure to the offending drug may cause new lesions to develop in addition to lighting up the older hyperpigmentation lesions [3, 4].

Many cases of FDE are reported in clinical practice today with several variants being described based on their clinical features and distribution of the lesions [2]. It has a reported worldwide prevalence of 2–5% and accounts for approximately 16–21% of all cutaneous drug eruptions with antimicrobial, anti-inflammatory, and anti-convulsive agents being the most common causative drugs [5].

Ivermectin via mass distribution has become essential for health campaigns in the elimination of two disfiguring and devastating tropical diseases: onchocerciasis (river blindness) and lymphatic filariasis [6]. Hundreds of millions of people in polyparasitized poor communities around the world are taking ivermectin to combat various diseases, making it a panacea for resource-poor countries.

FDE secondary to ivermectin is reported extremely rarely, emphasizing that there are few reports of this condition. Here we present a rare case of multiple FDE in a 75-year-old man following repeated ivermectin intake.

## Case presentation

A 75-year-old man from the South-West Region of Cameroon (an endemic zone for onchocerciasis) and of Bamileke ancestry presented to our clinic with skin lesions that had been evolving for over a year. The eruptions were first noticed a few hours after he took 12 mg of ivermectin (Mectizan) during mass drug administration (MDA) campaigns carried out every 3 months (as part of the public health strategy and in line with a recommendation from the World Health Organization’s African program for control of onchocerciasis [7] and to fight against filariasis in endemic parts of Cameroon). The initial eruptions were dark, itchy discolorations with occasional burning and appeared as single isolated rashes on his groin, genital, and neck regions.

On further inquiry, he described similar symptoms in the past whenever he took ivermectin which disappeared after he stopped the drug. Further consumption of ivermectin (2 months prior to consultation) during the ensuing campaign resulted in worsening of the old lesions with development of multiple new lesions over his face, back, and extremities. His family and medical history were not remarkable for any previous drug or cross-reactivity reactions.

**Fig. 1:**
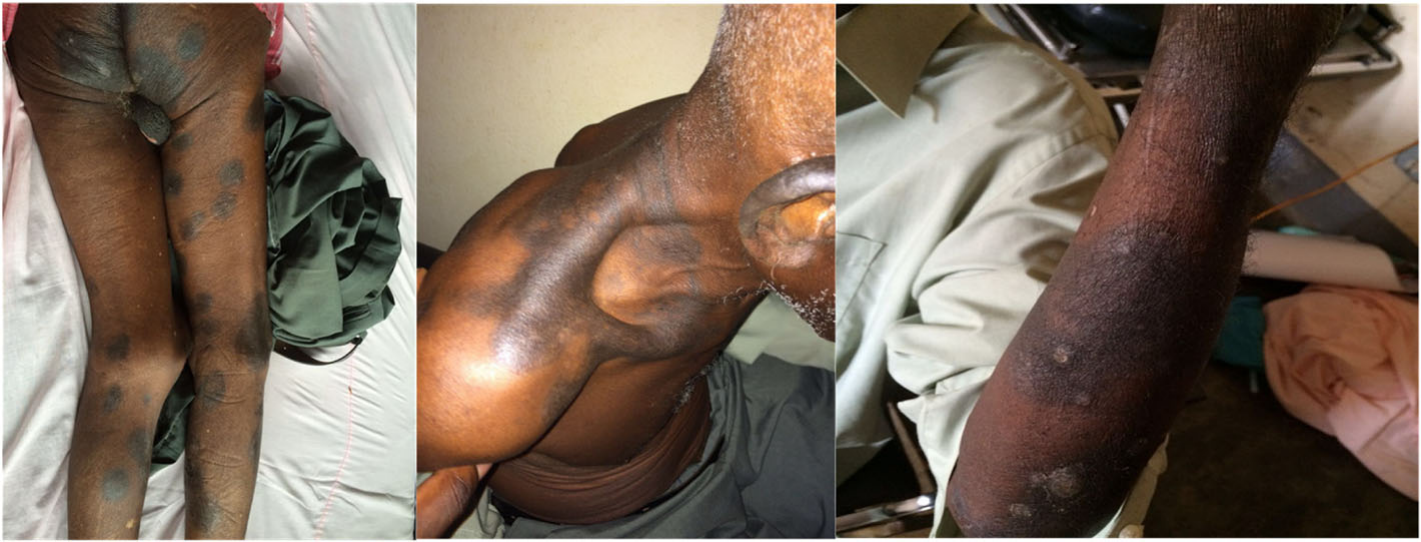
Multiple hyperpigmented annular plaque lesions on the shoulder, groin, and extremities

On physical examination, he looked well with vital signs within normal limits. There were multiple well-defined circular erythematous hyperpigmented plaque lesions of sizes ranging from 1 × 3 cm to 7 × 10 cm on his face, neck, groin area, and both extremities (Fig. 1) occupying approximately two-thirds of his total body surface area (TBSA). Other systemic examinations were normal.

A laboratory work-up including full blood count, human immunodeficiency virus (HIV) serology, urine analysis, and biochemistry (liver and kidney function tests) were normal. Erythrocyte sedimentation rate was at 65 mm/hour after the first hour, while punch biopsy of the skin, and antinuclear antibodies (ANA)/antineutrophil cytoplasmic autoantibody (ANCA) were requested but were unavailable.

A working diagnosis of FDE was made based on clinical signs and patient history despite the lack of histopathological findings.

**Fig. 2:**
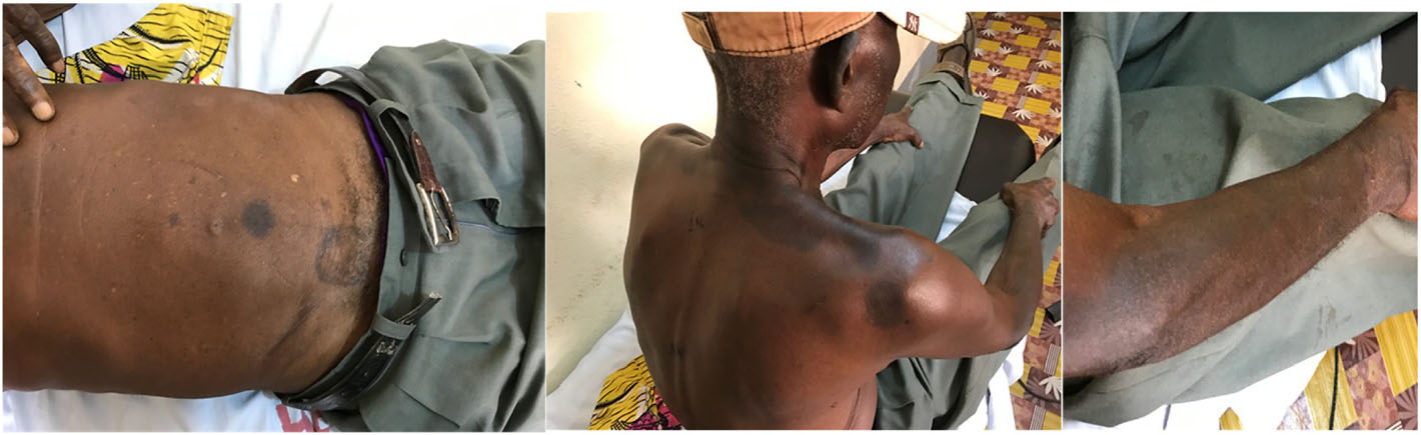
Resolution of lesions with residual hyperpigmentation and dyschromia

**Fig. 3:**
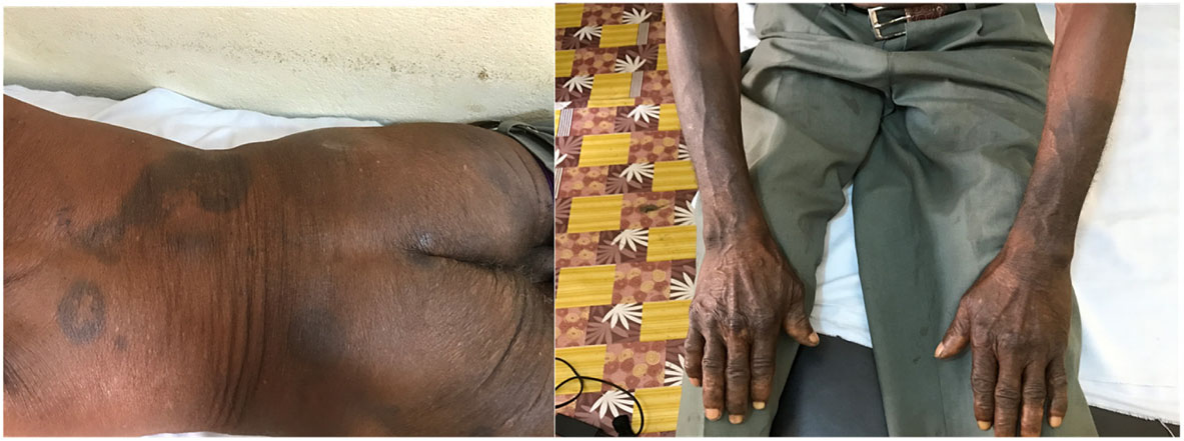
Resolution of skin lesions on back, gluteus area, and forearm

Discontinuation of ivermectin (plus counselling on avoidance of other possible culprits), a short course of systemic corticosteroids (prednisone 60 mg daily for a week), and orally administered antihistamines (hydroxyzine 75 mg daily) were employed as treatment modalities. Close patient follow-up revealed marked regression of lesions within a fortnight with residual hyperpigmentation (Figs. 2 and 3).

## Discussion and conclusions

Ivermectin, a semi-synthetic derivative of avermectin, was discovered in 1975 and is a broad-spectrum anti-parasitic compound with the ability to kill parasites both inside and outside the body; hence the term endectocide [8, 9]. It is recommended to treat onchocerciasis, cutaneous larva migrans, strongyloidiasis, scabies, and myiasis. Outside its anti-parasitic effects, it has also been shown to exhibit anti-bacterial, antiviral, and anti-neoplastic properties [8]. It consists of a mixture of two homologous compounds, which are 22,23-dihydroavermectin B1a (approximately 80%) and 22,23-dihydroavermectin B1b (approximately 20%), and it is radically different from other available compounds [10]. Onchocerciasis against which ivermectin is extremely effective, is considered to be a neglected tropical disease with an estimated 99% of all cases reported in sub-Saharan Africa and over 18 million people suffering from the disease [11].

Side effects from ivermectin are common, and include mostly abdominal pain as well as asthenia, hypotension, and dizziness. Prolonged liver dysfunction in a patient with scabies has been reported [12]. CADRs to ivermectin are not uncommon as Stevens–Johnson syndrome (SJS) has been reported in an immunocompromised individual [13]. Other drug eruptions include itchy papular rashes and bullous skin reactions; however, FDE is rarely reported in the medical literature.

Severe flares of FDE may closely resemble other forms of erythroderma including atopic dermatitis, erythema multiforme (EM), SJS, and hypersensitivity vasculitis, both clinically and histologically. Biopsy of skin specimens may be required to aid the diagnosis in selected situations.

Our patient reported lesions a few hours following ingestion of ivermectin and he had multiple sharply marginated, annular, itchy, erythematous plaques which would make FDE more likely than EM and SJS where lesions are atypical, irregular, purpuric macules with occasional blistering. This was further confirmed by the fact that discontinuation of the offending agent led to resolution of symptoms. The absence of palpable purpura, nodular lesions, ulceration, and racemosa coupled with no other system involvement made vasculitis less likely with the negative laboratory results.

Our diagnosis was based mainly on our patient’s history and we concluded that it was probable FDE based on the Naranjo Adverse Drug Reaction Probability Scale [14]: our patient had a cumulative score of + 7; adverse event occurring after suspected drug was administered and improving after discontinuation of the drug. He also reported having similar lesions to the same drug in the past and the adverse reaction reappeared when the drug was readministered (Table 1). Histologic findings of interface dermatitis with vacuolar change, Civatte bodies, dyskeratosis, and individual necrotic keratinocytes within the epidermis would definitively confirm the diagnosis of FDE [15]. Unfortunately, an absence of histopathology services, and, even more so, the financial constraints of our patient precluded us from investigating further. This is a common occurrence in low-income countries like ours, especially where there is an absence of universal health insurance and the cost of treatment is essentially paid by patients. The end result is physicians in these settings must use a syndromic approach in their management, and must rely on a high index of clinical suspicion and examination findings.

**Table 1:** Naranjo Adverse Drug Reaction Probability Scale

Questions	Yes	No	DK
1. Are there previous *conclusive* reports on this reaction?	+ 1	–	–
2. Did the adverse event appear after the suspected drug was administered?	+2	–	–
3. Did the adverse reaction improve when the drug was discontinued or a specific antagonist was administered?	+ 1	–	–
4. Did the adverse event reappear when the drug was re-administered?	–	–	0
5. Are there alternative causes (other than the drug) that could on their own have caused the reaction?	− 1	–	–
6. Did the reaction reappear when a placebo was given?	–	+ 1	–
7. Was the drug detected in blood (or other fluids) in concentrations known to be toxic?	–	0	–
8. Was the reaction more severe when the dose was increased or less severe when the dose was decreased?	+ 1	–	–
9. Did the patient have a similar reaction to the same or similar drugs in *any* previous exposure?	+ 1	–	–
10. Was the adverse event confirmed by any objective evidence?	+ 1	–	–

The ADR is assigned to a probability category from the total score as follows: ***definite*** if overall score is 9 or greater, ***probable*** for scores 5–8, ***possible*** for scores 1–4, and ***doubtful*** if the score is 0

*ADR* adverse drug reaction, *DK* do not know

An orally administered drug re-challenge test was not done on this patient as he denied consent, however, the fact that he reported lesions each time after the consumption of ivermectin made it the likely culprit here. Re-challenging the patient to the suspected offending drug is the only known test to possibly distinguish the causative agent, however, patch testing of suspected drug can also be helpful in checking cross-sensitivities to medications [16, 17].

Given the severity of this condition (approximately two-thirds of TBSA), a short course of systemic corticosteroids and orally administered antihistamines were administered alongside discontinuation of the drug with education on other possible culprits (mainstay of treatment). Follow-up was marked by regression of lesions with apparent dyschromia (resulting from the residual hyperpigmentation).

We have presented, to the best of our knowledge, a very rare case of FDE secondary to ivermectin from Cameroon. Although a non-life-threatening complication of CADRs and bearing in mind that other life-threatening complications like SJS have been recently reported [13], the general population and especially health personnel should not only be aware but increase vigilance during mass drug campaigns for such reactions.

## Abbreviations

ANA: Antinuclear antibodies
ANCA: Antineutrophil cytoplasmic autoantibody
CADR: Cutaneous adverse drug reaction
EM: Erythema multiforme
FDE: Fixed drug eruption
HIV: Human immunodeficiency virus
MDA: Mass drug administration
SJS: Stevens–Johnson syndrome
TBSA: Total body surface area
